# Insect Gut Microbiota—Research Strategies and Perspectives

**DOI:** 10.3390/insects17040367

**Published:** 2026-03-30

**Authors:** Xinyu Li, Zhen Ye, Shangling Wu, Yan Lv, Yinzheng Ren, Qin Luo, Hong Yang

**Affiliations:** Key Laboratory of Pesticide & Chemical Biology of Ministry of Education, Hubei Key Laboratory of Genetic Regulation and Integrative Biology, School of Life Sciences, Central China Normal University, Wuhan 430079, China; leexinyu@mails.ccnu.edu.cn (X.L.); yezhen@mails.ccnu.edu.cn (Z.Y.); wushangling@mails.ccnu.edu.cn (S.W.); 19872020949@mails.ccnu.edu.cn (Y.L.); yinzhengren1026@163.com (Y.R.); qinluo@ccnu.edu.cn (Q.L.)

**Keywords:** insect, gut microbiota, functions, culture-dependent, culture-independent

## Abstract

Insects are widely distributed across the globe and exhibit strong adaptability in diverse living environments, a capability closely linked to the diversity of their gut microbiota. The composition of insect gut bacteria varies with species, living environment, diet, and development stage. In recent years, the widespread application of culture-independent strategies based on molecular biology techniques has provided substantial information for studies on the interaction mechanisms between insects and their gut microbiota. However, culture-dependent strategies aimed at isolating pure cultures remain indispensable. Only by integrating multi-techniques such as bacterial isolation and pure culture, axenic insect technology, and molecular biology can in-depth research be conducted on key gut bacteria of insects. This review summarizes culture-dependent and -independent strategies used for the analysis of the diversity and functions of insect gut microbiota, focusing on the traditional methods and new strategies for microbial cultivation, multi-omics techniques, and axenic insect technology. Recent studies showed that the application of integrated techniques is powerful for illustrating the microbial function and evolution of gut microbiota, and the interactions between intestinal bacteria and their hosts. Studies have shown that the insect gut microbiota plays important roles in the promotion of host growth and development by regulating host metabolic pathways, contributing to host nutrition, and supporting the host in defending against pathogens or degrading toxic compounds. Future research directions and strategies are also proposed, providing insights into further exploration of the interaction mechanisms between symbiotic insect gut bacteria and their hosts, as well as future applications in various fields.

## Simple Summary

The gut microbiota of insects enhances their adaptability and fitness in various environments. The diversity of the intestinal microbiota of insects is influenced by many factors such as the host’s species, dietary habits, gut physiochemical conditions, environment, and life cycle. Studies have shown that the gut microbiota plays various important roles in the growth and development of insects, such as providing nutrition, resisting pathogens, and degrading toxins. Research on the insect gut microbiota and its interactions with hosts holds great significance for future applications. This review summarizes culture-dependent and -independent strategies used for the analysis of the diversity and functions of insect gut microbiota, discusses the research progress of axenic insect technology, and summarizes recent research findings on the functions of the gut microbiota of insects based on integrated research approaches. This review also emphasizes the significance of axenic insect technology in studying the interactions between insect gut bacteria and hosts, with the aim of providing insights for future research related to insect gut microbiota.

## 1. Introduction

Insects are the most diverse and abundant animals on Earth. The adaptation of insects to distinct environments has depended partially on their close relationships with the gut microbiota [1]. Over the course of 480 million years of evolutionary history, insects have formed diverse symbiotic relationships with intestinal microbiota, including bacteria, archaea, fungi, protozoa, and viruses [2,3]. The microbiota accounts for 1–10% of the insect’s biomass and plays important roles in nutrition, immunity, toxic compound degradation, etc. [4]. Therefore, the microbiota impacts insects’ growth and development; fitness; and microbe-mediated behavior, such as disease transmission by mosquitoes [5,6]. With the development and application of high-throughput sequencing techniques, the diversity and community structures of a large number of insect microbiomes have been reported in recent decades. Studies have shown that a variety of factors may affect the composition of insect gut microbiota, including species, habitat, diet, genetic factors, and developmental stages, which have been reviewed exhaustively by many researchers [7,8,9,10,11].

Although the microbial diversity of many insects has been revealed, our understanding of the functions of gut microbiota is still limited. The exploration of the functions of symbiotic microbiota relies on pure cultured microorganisms. However, most of the symbiotic microorganisms are difficult to cultivate, which has hampered the study of their functions [12]. In recent decades, culture-independent techniques such as high-throughput sequencing and multi-omics combined with bioinformatic analysis have been applied to demonstrate the diversity and functions of symbiotic gut microbiota, which greatly promote the development of this field. In this review, we summarize the progress in multiple techniques, including culture-dependent and -independent techniques, as well as axenic insect technology. The application of integrated techniques is highlighted with selected case study examples to show the recent progress in the functional analysis of symbiotic microorganisms, providing insight for future studies on the theories of insect–microbe interactions and the application of insect gut microorganisms in fields such as agriculture, health, and environmental management.

## 2. Culture-Dependent Techniques

### 2.1. Insect Gut Physiology and Its Impact on Microbial Community Structure

Insects have similar digestive tract structures, although there are differences among various species. The insect gut is divided into three distinct regions based on embryonic derivation, morphology and function. The foregut and hindgut originate from the embryonic ectoderm and are lined with an exoskeleton that is shed at each ecdysis, whereas the midgut originates from the embryonic endoderm. The foregut consists of the pharynx, esophagus, crop, and gizzard and is responsible for transporting and temporarily storing food. Some of this food is primarily digested with the enzymes secreted by the salivary glands. For most insect species, the midgut is the largest region of the intestine and plays a crucial role in food digestion and nutrient absorption [1]. In many insects, the midgut epithelial cells secrete an envelope called the peritrophic matrix that prevents the direct contact of food with the midgut epithelium, thus forming a barrier to protect the epithelium [13]. The hindgut is the posterior section of the intestine in insects. Its main functions are water absorption and feces formation, except in termites and wood-feeding cockroaches in Blattaria, whose hindgut is enlarged to a big paunch and is the major place for food digestion [2].

The physical conditions in the lumen of different gut regions of various insects are diverse in pH, oxygen concentration and redox potential, which greatly influence the microbial community structure. The pH of the lumen is regulated and often diverges from that of the hemolymph, which is usually near 7. However, the midguts of lepidopteran larvae are extremely alkaline, with pH as high as 11–12, and digestive enzymes are adapted to the alkaline conditions [14,15]. In the hindgut of wood-feeding higher termites *Nasutitermes corniger* (Blattodea: Termitidae) and soil-feeding higher termites *Cubitermes* spp. (Blattodea: Termitidae), the pH was found to be acidic to neutral in the midgut, 9–12 in the frontal hindgut parts P1–P3, and neutral in the posterior parts P4–P5, which are adapted to the digestion of various food contents [16,17]. The pH profile of the intestinal tract of Diptera insects is also complicated. One example is the pH values of different gut lumens in the midgut of the black soldier fly (BSF), *Hermetia illucens* (Diptera: Stratiomyidae) larvae. The pH values change from weak acid (pH < 7.0) in the anterior midgut to strong acid (pH < 3.0) in the enlarged middle midgut, then become nearly neutral to alkaline (pH > 8.0) in the posterior midgut, and the bacterial community structure changes drastically with the divergence of gut physiochemical conditions [18].

In addition to pH values, oxygen/hydrogen concentrations and redox potential may be quite different along the intestinal tract. In the hindgut of the eastern subterranean termite *Reticulitermes flavipes* (Blattodea: Rhinotermitidae) (a lower termite), there is a steep radial gradient of oxygen partial pressure from the highest levels in the gut epithelium to zero in the center of the hindgut lumen, whereas the reverse is observed for hydrogen partial pressure [19]. A radial gradient of oxygen partial pressure is also present in the freshwater moth *Acentria ephemerella* (Lepidoptera: Pyralidae). Oxygen concentrations decrease linearly from the gut epithelium to the center of the midgut, identifying the midgut as an oxygen sink [20]. In the wood-feeding higher termites *N. corniger* and soil-feeding higher termites *Cubitermes* spp., the redox potential of the hindgut P3 region is below −200 mV, whereas the values of the midgut and other regions of the hindgut are 0 ~ +400 mV [17].

Corresponding to axial differences in pH, redox potential, oxygen, and hydrogen concentrations of the *Cubitermes* hindgut, distinct bacterial and archaeal compositions are present in different hindgut compartments [16,21,22,23]. For the soil-feeding termite *Cubitermes orthognathus* (Blattodea: Termitidae), the main Archaeal group in the extremely alkaline frontal part (P1) is Methanosarcinaceae, whereas Methanobacteriaceae and Methanomicrobiales are dominant in the neutral part (P3 and P4), and Thermoplasmales are dominant in the acidic posterior part (P5) [22]. For the soil-feeding termite *Cubitermes ugandensis* (Blattodea: Termitidae), the major bacterial group in the extremely alkaline P1 compartment is Firmicutes with low GC content. In addition to Firmicutes, Bacteroides and Planctomycetales are the dominant groups in the neutral P3/P4 parts and acidic P5 part, respectively [23]. Further study on the wood-feeding termite *Reticulitermes santonensis* (Blattodea: Rhinotermitidae) revealed the niche heterogeneity of the intestinal tract-determined bacterial community structure [24]. In the hindgut wall, where oxygen concentration is high, the major bacterial groups are Firmicutes and Bacteroides, whereas Enlusimicrobiota (previously called TG1) is dominant in the anaerobic center of the hindgut lumen.

### 2.2. Techniques for the Cultivation of Insect Gut Microorganisms

Many gut microorganisms have been isolated from insect intestinal tracts using traditional culture techniques [1]. The most commonly used techniques are the streak plate method, or serial dilution, and the spread plate method. Both are based on the dilution-to-extinction principle so that single bacterial colonies can be obtained and isolated from mixed bacterial populations. Different media and conditions (aerobic, facultative anaerobic, or strict anaerobic) were used for the cultivation of various microbes depending on the physiochemical conditions of insect guts. Under aerobic conditions, many aerobic and facultative anaerobic symbiotic bacteria have been isolated from insect guts, such as *Enterococcus* sp. from the spongy moth *Lymantria dispar* (Lepidoptera: Erebidae) [25], *Lactobacillus kunkeei* from honey bees, and *Lactococcus nasutitermitis* from termites [26,27]. Strict anaerobes such as *Methanobrevibacter cuticularis* and *Methanobrevibacter curvatus* have been successfully cultivated from *R. flavipes* under anaerobic conditions [28].

To obtain specific gut symbiotic bacteria, the enrichment-based cultivation approach has been frequently applied using different selective media under appropriate conditions to mimic the microbial niches of the targeted insect gut microbes. Enrichment culture is a commonly used method for isolating bacteria. By providing nutrients or environmental conditions preferred by the target bacteria, it enables the target bacteria (originally present in low proportions in the sample) to proliferate rapidly and become the dominant species in the mixed culture by restricting the growth of nontarget bacteria. This technique often works synergistically with a selective medium [29]. Using cellulose as the sole carbon source, *Clostridium termitidis* was enriched and isolated from the gut of *Nasutitermes lujae* (Blattodea: Termitidae) under strict anaerobic conditions [30]. Many cellulolytic bacteria such as *Paenibacillus* and *Bacillus* were isolated from the gut of Lepidoptera insects *Spodoptera frugiperda* (Lepidoptera: Noctuidae) and *Diatraea saccharalis* (Lepidoptera: Crambidae), after enrichment in media supplemented with sugarcane bagasse or sodium carboxymethyl cellulose [31]. To enrich spirochaetes in the hindgut of *Zootermopsis angusticollis* (Blattodea: Termopsidae), Leadbetter and coworkers established an enrichment culture with a medium containing rifamycin and phosphomycin (two drugs to which many spirochetes are resistant) under anaerobic conditions with a headspace of H_2_ plus CO_2_. The medium also contained bromoethanesulfonate to inhibit the growth of H_2_-consuming methanogens [32]. Using specific media without a nitrogen source, an azotobacter *Endomicrobia proavitum* with type IV nitrogenase was isolated from the gut of *R. santonensis* [33]. A mixed culture including archaea *Thermoplasmatales* as the dominant component was enriched from the gut of *Cubitermes ugandensis* (Blattodea: Termitinae) with nitrogen-free medium. An analysis of the 16S rRNA gene and *mcrA* gene confirmed the nitrogen-fixation activity of the enrichment culture [34]. Although many new symbiotic microorganisms have been isolated in recent years, compared with the microbial diversity revealed by culture-independent techniques based on 16S rRNA gene sequencing, only a very small portion of the insect intestinal bacteria have been successfully cultivated [2], leaving a huge gap for the discovery of eco-physiological functions of symbiotic microbes inhabiting insect guts.

New techniques and strategies have been developed in recent years to gain more cultivable bacteria from insect guts. One of the strategies is to use oligotrophic media to cultivate microbes in low-nutrient environments. The traditional media such as Luria–Bertani (LB) and Tryptic Soy Broth (TSB) are rich in nutrients that inhibit the cultivation of many slow-growing microbes. Diluting these media to 3-fold, 5-fold, or 10-fold and applying them for microbe cultivation can simulate nutrient-poor conditions and increase the cultivable bacteria that normally grow slowly in low-nutrient environments [35,36]. Based on this principle, new oligotrophic media such as AM-4 medium and MM-4 medium were designed for the isolation of symbiotic bacteria from insect guts [37]. Using oligotrophic media 1/5 LB, 1/3 TSB, and MM-4, bacteria affiliated with 17 genera were isolated from the gut of *Reticulitermes chinensis* (Blattodea: Rhinotermitidae), among which six bacterial strains were identified as possible new species at species or genus levels, including bacteria belonging to the genera *Lactococcus*, *Deinococcus*, and *Gryllotalpicola* and the family Opitutaceae [27,38,39]. Some bacteria, such as those affiliated with the genera *Deinococcus* and *Gryllotalpicola,* were first isolated from the gut of termite species, indicating that the application of oligotrophic media is helpful for the cultivation of the so-called “uncultivable” bacteria. In addition to the use of oligotrophic media, supplementing specific nutrients such as vitamins and extending incubation time also help to increase the ratio of cultivable bacteria from different environments [40,41,42].

In recent decades, high-throughput cultivation strategies have been developed. Stevenson and coworkers developed a simple, high-throughput, PCR-based technique, the so-called plate wash PCR (PWPCR), to isolate specific bacteria from different environmental samples such as soil and termite gut [43]. The key stages of PWPCR are as follows: (1) inoculate microbial samples and incubate them on agar plates with different media and conditions; (2) wash different bacterial colonies from the agar surface with fresh buffer and perform PCR with group-specific primers; and (3) screen target bacteria and transfer the colonies to fresh media for further incubation. This method greatly facilitates the detection and ultimate isolation of target bacteria from as many as 1000 colonies of nontarget microbes growing on the same agar plates. Using this technique, five different Verrucomicrobiota strains were successfully isolated from the wood-feeding termite, *R. flavipes*, for the first time [43].

More recently, microfluidics-based cultivation methods have been developed [44,45]. This technique enables the manipulation of fluids at the microscale with microchannels at sizes of tens to hundreds of microns [46]. Droplet-based microfluidics is one of the most developed techniques for high-throughput single-cell cultivation. Combined with different culture media and conditions, it greatly promotes the cultivation of uncultivable microorganisms [47]. The newly cultivated microbes can be further identified with next-generation sequencing (NGS), which is powerful for the characterization and functional mining of microbiota in different environments including insect guts [48]. One example is the microfluidic streak plate technique (MSP) [45]. This technique exploits the advantages of microfluidics to manipulate a tiny volume of liquid at several hundred nanoliters and generate microdroplets for single-cell microbe isolation and cultivation. The first step of MSP is to generate nanoliter droplets using microfluidic devices. Then, the droplets are streaked manually or robotically onto Petri dishes prefilled with carrier oil for culturing single cells. MSP offers higher throughput than traditional cultivation methods, as one plate can accommodate thousands of droplets. With this technique, 99 operational taxonomic units were cultivated from the gut of *R. chinensis*, among which potential new taxa from the genera *Burkholderia*, *Micrococcus*, and *Dysgonomonas* at species and/or genus levels have been successfully cultivated [49].

Another example is the construction of a picodroplet microfluidic platform. This platform can generate ~6 × 10^8^ droplets encapsulated with individual bacterial cells and cultivate them in different media. The droplet volumes are around 14 μL, and the bacterial suspension is incubated with 1 μg/mL DAPI stain solution before the generation of droplets. Each droplet with a single cell is incubated with culture medium and then visualized using an inverted fluorescence microscope. In this way, the morphology and status of growing bacteria can be observed directly. Shotgun metagenomic analysis is applied to each bacterial isolate. This can help to elucidate the inter-strain interactions and strain-level diversity through comparative analysis of metagenome-assembled genomes. The application of this new strategy revealed that there is a high strain-level diversity of symbionts such as *Bifidobacterium* sp. and *Lactococcus panisapium* in the gut of honey bees. Comparative genome analysis showed that different strains might harbor enzymes belonging to various GH families, indicating high adaptation to polysaccharide degradation of the host [48].

### 2.3. Whole-Genome Sequencing

Whole-genome sequencing (WGS) is a DNA sequencing technique where the entire genome is randomly broken into small fragments, which are then sequenced and assembled using computational algorithms to reconstruct the full genome [50]. Sequencing can be performed either with universal primers after the fragments are repaired and cloned, or by end-repairing the fragments and adding amplification and sequencing tags specific to each sequencing platform. It can uncover the complete genomic information of a single microorganism and represent an unbiased sequencing strategy. WGS yields genome-based species identification results with the highest resolution, which can predict the functional capabilities of isolated strains such as antibiotic resistance, organic matter decomposition, and pathogenesis [51]. Further experiments can be performed to test the predicted functions of the isolated strains. By conducting whole-genome sequencing and analysis on strains isolated from the intestines of various insects such as the *Periplaneta americana* (Blattodea: Blattidae), bees, and silkworms, researchers have discovered that these strains possess distinct functional genes, such as those encoding different carbohydrate enzymes or those involved in the synthesis of specific amino acids (e.g., L-tryptophan) [52,53,54].

## 3. Culture-Independent Techniques

### 3.1. Investigation of Microbial Diversity Based on SSU rRNA Gene Sequencing

The development of molecular phylogeny based on marker genes such as SSU rRNA has greatly enhanced our understanding of microbial diversity in different environments since the nineteen-nineties, and overcomes the restrictions and potential biases of cultivation methods [55]. The most commonly used marker genes are SSU rRNA genes, namely 16S rRNA genes of Bacteria and Archaea and 18S rRNA genes of Eukaryotes. The 16S rRNA/18S rRNA genes are DNA sequences corresponding to the small subunit of ribosomes in prokaryotic/eukaryotic cells. They exist in the genomes of all prokaryotic/eukaryotic cells and exhibit high conservation and specificity. PCR amplification and sequencing of the SSU rRNA region, combined with analysis based on existing databases, enable inference of the taxonomic information of the microbial diversity in a sample. Since the application of this molecular phylogeny technique, the microbial communities in various environments, such as insect and human guts, soil, and marine sediments, have been elucidated in numerous studies [17,56,57,58]. Further comparative analysis with cultured bacteria showed that the nearly complete 16S rRNA sequences give accurate measures of taxonomic diversity [59].

With the fast development of sequencing techniques, high-throughput sequencing (HTS) based on SSU rRNA gene amplicons is widely used to determine the intestinal microbial diversity of various insects such as termites, honey bees, and mosquitoes [26,60]. This technique is low-cost, with mature data analysis pipelines and a large volume of archived data available for reference [56,57,58]. It is frequently used for the analysis of the dynamic changes in the gut microbiota of insects in different life cycles, such as the BSF and the bumble bee, *Bombus lantschouensis* (Hymenoptera: Apidae) [61,62], or in different seasons, such as the Eri silkworm, *Samia ricini* (Lepidoptera: Saturniidae), and the honey bee [63,64].

### 3.2. Meta-Omics Techniques

Due to the rapid development of sequencing techniques and biochemical techniques in the last two decades, many high-throughput techniques such as metagenomics and metatranscriptomics have been established and widely used for exploring microbial diversity and functions in various environments.

Metagenomics and de novo assembly based on bioinformatic analysis have yielded rich collections of metagenome-assembled genomes (MAGs) of microorganisms such as bacteria, archaea, fungi, and viruses [52,65,66]. By deciphering DNA sequence information, these techniques have revealed the diversity, taxonomic status, and functional potential of microbial communities (e.g., genes encoding specific enzymes in metabolic pathways). The development of metagenomic sequencing is a landmark breakthrough, leading to the discovery of many new bacterial and archaeal species in different environments, making it possible to explore the ecological functions and evolution of “uncultivable” microbes in nature [67]. With this technique, 67 metagenome-assembled genomes (MAGs) of *Endomicrobiaceae* were obtained from the gut microbiota of a wide range of termites, most of which were important endosymbiotic bacteria associated with cellulolytic protozoa in wood-feeding lower termites [68]. Comparative genome analysis documented progressive genome erosion in the new genus *Endomicrobiellum*, which comprises exclusively endosymbiotic bacteria living in the cytosol of cellulolytic flagellates, indicating their obligate mutualistic relationship with flagellates.

Metatranscriptomics is a high-throughput sequencing technique based on cDNA. It is used for the analysis of the whole-genome expression profiles by extracting mRNA from samples, then reverse-transcribing it into cDNA and subsequently conducting sequencing [69]. It reveals the genes expressed instantaneously under specific conditions and their associated functions. Metatranscriptomics exhibits a sensitive response to physiological fluctuation; it can distinguish active microorganisms and reflect real-time functional activity. The research object of conventional transcriptomics is the entire transcriptome of a single organism/cell/species under specific conditions, while metatranscriptomics analyzes the complex microbial communities in the environment, focusing on the overall gene expression at the population level [70]. Metatranscriptomics is often used to mine novel genes with biotechnological value (e.g., enzyme-encoding genes) from various environments, analyze the functions of microbial communities, and clarify the expression patterns of different genes in insects under different statuses. A total of 68 differentially expressed genes were identified through comparative analysis of the transcriptomic profiles of brain samples from conventionally reared, microbiota-free, and mono-colonized honey bees. Subsequent metabolomic analyses revealed that host-specific *Lactobacillus* strains promote memory behavior by transforming tryptophan to indole derivatives and activate the host aryl hydrocarbon receptor [71]. In a study on the cellulose degradation mechanisms of symbiotic bacteria of the wood-feeding higher termite *Nasutitermes takasagoensis* (Blattodea: Blattidae), metatranscriptomic analysis was conducted on fiber-associated bacteria in the hindgut [72]. The results showed that the degradation of xylan, the major component of hemicellulose, is restricted to the hindgut. The most abundantly expressed glycoside hydrolase (GH) is a GH11 family enzyme that was found to be exclusively responsible for xylan degradation. Moreover, it was confirmed that the most dominant GH11 members were primarily encoded by bacteria of the phylum *Spirochaetes*, thus demonstrating that fiber-associated spirochetes are the core microbial group responsible for xylan degradation in the hindgut of *N. takasagoensis*.

Proteomics is a technique that aims to uncover the identities, quantities, structures, interactions, and modifications of the complete set of proteins expressed in cells, tissues, or organisms to reveal their biological functions [73]. The core protein detection technology is liquid chromatography–tandem mass spectrometry (LC-MS/MS), and the shotgun proteomics method is commonly used for analysis. Metaproteomics is an emerging approach for studying microbiomes, offering the ability to characterize proteins that reflect microbial functionality within diverse ecosystems. This is a technique for characterizing all the products of gene translation, and it provides additional information about post-translational modifications and localization information over transcriptome measurements [74]. Combined with metagenomics and metatranscriptomics, it can reveal which community members are active and involved in specific biological processes under a particular ecological system, such as the gut of the weevil *Cryptorhynchus lapathi* (Coleoptera: Curculionidae) [75]. In this study, the whole gut was dissected from larval weevils, and the anal droplets were collected. 16S rRNA sequencing was performed to analyze bacterial community structure, while metaproteomics was used to explore the functions of microbiota. The results showed that the dominant bacteria were different between the gut lumen and the anal droplets. Metaproteomics data indicated that the most important role of gut bacteria is amino acid biosynthesis, followed by protein digestion, energy metabolism, vitamin biosynthesis, lipid digestion, and plant secondary metabolite (PSM) degradation. Metaproteomics is also used to study pesticide resistance in insects [76]. Phoxim is a commonly used pesticide against the onion maggot *Delia antiqua* (Diptera: Anthomyiidae), and high resistance has been demonstrated. It was shown through comparative metaproteomic analysis of gut samples from resistant and sensitive *D. antiqua* strains that proteins from both host and gut microbiota made contributions to phoxim degradation and resistance.

Metabolomics is a technology used to perform high-throughput qualitative and quantitative analysis of metabolites (carbohydrates, lipids, amino acids, nucleotides, etc.) with a molecular weight of less than 1500 Da, and it can reflect the state of biological systems. Its core techniques include nuclear magnetic resonance (NMR) and mass spectrometry (MS) combined with other techniques—liquid chromatography–mass spectrometry (LC-MS), gas chromatography–mass spectrometry (GC-MS), and ultra-high-performance liquid chromatography–mass spectrometry (UHPLC-MS)—which are applied for the qualitative and quantitative analysis of metabolites. Metabolomics can be generally divided into two categories: targeted and untargeted [77,78]. Targeted metabolomics focuses on known specific metabolites and is used for verifying results, evaluating the research value of biomarkers, and accurately detecting specific metabolites. Untargeted metabolomics is designed to capture all detectable metabolites in samples without preset targets; it is utilized to discover novel biomarkers, screen metabolic pathways, and explore unknown metabolic perturbations. Comparative analyses of metabolomic profiles between axenic *Drosophila suzukii* (Diptera: Drosophilidae) and those recolonized with the gut bacterium *Klebsiella oxytoca* revealed that the bacterium markedly remodeled the host’s metabolome by regulating carbohydrate metabolism in *D. suzukii* and modulating metabolite turnover in carbohydrate metabolic pathways [79]. Metabolomic analysis revealed that the key gut symbiotic bacterium *Klebsiella michiganensis* BD177 can activate the arginine–proline metabolic pathway in *Bactrocera dorsalis* (Diptera: Tephritidae), regulate key metabolites, and help the insect survive adverse low-temperature environments [80].

## 4. Axenic and Gnotobiotic Insect Technologies

### 4.1. Axenic Insect Technique

Different insect guts harbor distinct bacterial communities. To analyze the functions of insect gut bacteria, it is necessary to obtain pure cultures of single strains of the key gut bacteria and minimize interference from low-abundance microorganisms. Axenic insect technology can eliminate the influence of the gut microbiome on insects, which contributes to a deeper understanding of the impact of symbionts on their hosts. Effective sterilization, a nutritionally adequate diet, and rearing insects under a controllable environment are important for establishing an axenic insect system [81]. Typically, the establishment of germfree (GF) insects begins with a specific period when axenic individuals can be easily obtained. For example, the spread of symbiotic bacteria in many insects occurs by applying microbial communities to the surface of the eggs. Therefore, sterile eggs can be obtained by removing surface microorganisms, and axenic insects can be produced from these eggs, allowing germfree insects such as the fruit fly *Drosophila melanogaster* (Diptera: Drosophilidae), BSF, mosquito, and cockroach to be obtained through this method [60,61,81]. Alcohol and sodium hypochlorite have been used for egg disinfection [60]. Another period is the pupal stage, during which insects undergo a process of gut bacteria reorganization. The pupal stage of bees involves a “molting” phase; the inner lining of the gut is shed, removing all bacteria in the midgut. Axenic adult bees can be obtained by cleaning the pupae and placing them in a sterile environment [82].

Antibiotics are widely used in studies on the interaction between insect symbiotic bacteria and their hosts, which reduces the complexity of the insect gut microbiota. Chen and coworkers used different antibiotics to induce gut dysbiosis in order to investigate the possible mechanisms of gut microbial resistance to insecticides in the silkworm *Bombyx mori* (Lepidoptera: Bombycidae) [83]. Both Penicillin G and Polymyxin B can significantly reduce the total number of gut bacteria in silkworms, and Polymyxin B, in particular, markedly decreases the abundance of *Stenotrophomonas*. Further experiments with reinoculated GF silkworms proved that *Stenotrophomonas* plays important roles in host resistance against the pesticide chlorpyrifos. After culturing the small brown planthopper *Laodelphax striatellus* (Hemiptera: Delphacidae) with tetracycline for five generations, the abundance of *Firmicutes*, *Bacteroidetes*, *Tenericutes*, and *Fusobacteria* in the midgut decreased significantly; meanwhile, *Wolbachia*, *Bacteroides*, and *Abiotrophia* were almost eliminated [84]. However, it should be noted that since antibiotics may interfere with the growth, development, and reproduction of insects, they are generally only used as an auxiliary measure in aseptic techniques [81,85,86]. Autoclaving, radiation, and filtration are common approaches for obtaining sterile food [83,87].

Axenic systems also require regular checks of the aseptic status of insects and the environment. The presence of bacteria in samples is generally detected using 16S rRNA gene PCR and inoculation of the samples into the culture medium for cultivation [57,88]. The success rate of obtaining axenic insects from different treatment methods varies, and maintaining an aseptic system requires strict control over sterilization, substrates, and growth environments.

### 4.2. Gnotobiotic Insect System

Gnotobiotics describes the established association of axenic organisms with one or more known microbial species [81]. To understand the functions of a certain microbe on its host, gnotobiotic insects are reared by artificial introduction of one or more known bacteria into the axenic insects. Target microbes added to the growth environment of the larvae allow sterile larvae to come into contact with the bacterial community [61,84,89]. There are three main approaches for introducing microorganisms in axenic insects: (1) Food supplementation—target microorganisms (either a single strain or a mixed microbial consortium) are mixed into the diet of germfree insects, allowing the microorganisms to colonize the host naturally through ingestion [90,91]. (2) Direct injection—microbial suspension is delivered directly into the insect body via microinjection technology to ensure rapid microbial colonization [81]. (3) Somatic or egg surface inoculation—this simulates the natural transmission process of bacteria that are transmitted via egg surfaces or physical contact, thereby achieving the colonization of either a single bacterial strain or a mixed microbial community [92]. Gnotobiotics relies on germfree insects as carriers and elucidates host–microbe interaction mechanisms by precisely introducing known microorganisms. This technology fills the research gap regarding the differences between axenic insects and conventional insects and enables a deeper understanding of the relationship between gut microbiota and the host. However, due to the lack of representativeness in its microbial composition, gnotobiotics cannot fully simulate the complex microbial interaction networks in natural environments. In addition, microbial colonization efficiency is affected by factors such as strain type, inoculation dose, and insect developmental stage, and it is difficult to achieve long-term stable colonization for some exogenous microorganisms.

GF and gnotobiotic insects are frequently used for the functional analysis of gut microbes. Predaceous *Toxorhynchites amboinensis* (Diptera: Culicidae) preys on the larvae of *Aedes aegypti* (Diptera: Culicidae). The first-instar larvae of *T. amboinensis* fail to develop when fed with germfree prey, while in larvae fed with gnotobiotic prey (prey colonized by one or more known bacterial species), the bacteria can colonize the gut of *T. amboinensis* and rescue its development [91]. Through 16S rRNA gene sequencing, axenic larval preparation, and intestinal bacterial recolonization, it was found that the axenic larvae of three mosquito species, *Ae. aegypti*, *Anopheles gambiae* (Diptera: Culicidae), and *Georgecraigius atropalpus* (Diptera: Culicidae), failed to develop beyond the first instar [56]. In contrast, inoculation with different bacteria—including the mosquito gut bacteria and non-mosquito gut bacteria such as *Escherichia coli*—enabled the larvae to develop into adults.

### 4.3. Microbiome Transplantation Technique

Microbiome transplantation is an approach in which the entire microbiota is extracted from donor insects (rather than a single strain), subjected to standardized treatment, and then transferred to germfree insects to recapitulate the natural process of microbiota colonization [93]. It is often used to explore host–microbe interactions and identify microbial taxa and assemblages associated with health or disease. Gut microbiome transplantation can be performed not only within the same species but also between different species [93]. For the study of mosquito–microbe interactions, Coon and coworkers developed an approach to transfer entire microbial communities either between two different mosquito species (*Culex quinquefasciatus* to *Aedes aegypti*) or within the same species (*Ae. aegypti* to *Ae. aegypti*). Fecal microbiota transplantation (FMT) is a procedure used for therapy in which processed stool is transferred from a healthy donor to a recipient’s gastrointestinal tract. The primary aim is to restore the balance of the gut microbiota. A study on honey bees showed that the composition of fecal microbiota is strikingly similar to the entire gut microbiota [94]. Through fecal microbiome transplantation, the experiment indicated that the contact of young bees with fecal matter in the hive might be the key pathway for gut microbiome transmission among nestmates.

## 5. Genetic Modification of Insects and Symbiotic Microorganisms

### 5.1. RNA Interference

RNA interference (RNAi) is a technique in which proteins of the Argonaute (Ago) family form a complex with small RNAs such as siRNA and miRNA, which specifically inhibit the transcription, stability, or translation of target genes, thereby reducing gene products or attenuating their functions [95]. By means of RNAi technology, it is possible to elucidate the functions of genes associated with insect development, reproduction, behavior, and immune responses. RNAi is also commonly used to identify insecticide target sites, clarify their mechanisms of action, and characterize key genes involved in pesticide resistance in insects. In insect-related research, common RNAi strategies mainly include basic delivery methods such as microinjection, feeding, and soaking, as well as nanoparticle- and microbe-mediated technologies developed to overcome delivery barriers. In terms of molecular design, silencing efficiency is often improved by optimizing dsRNA structure, co-silencing dsRNases, or enhancing the activity of RNAi pathways.

RNAi can be applied by directly targeting insect genes or indirectly downregulating insect genes by genetically modified symbiotic gut bacteria. By silencing the key factors of the Imd and Toll pathways in *Ae. aegypti* via RNAi and performing viral infection experiments, researchers found that the Toll pathway plays a critical role in regulating resistance to dengue virus [96]. In a study of the function of the BSFL gut symbiotic bacterium *Citrobacter amalonaticus* CABG02, researchers developed a symbiont-mediated double-stranded RNAi approach by introducing a plasmid defective in ribonuclease III (RNase III) to *C. amalonaticus* CABG02; it was shown to successfully achieve stable gene knockdown in larval midgut in situ. Ultimately, it was demonstrated that *C. amalonaticus* CABG02 enhances larval protein digestion by upregulating the Hitryp serine protease and Himtp metallopeptidase families [97].

Ding and coworkers also developed a symbiont-mediated RNA interference (RNAi) technique for the control of mosquitoes [98]. By knocking out the RNase III gene of the symbiotic bacterium *Serratia* sp., they constructed an engineered strain capable of stably producing double-stranded RNA (dsRNA). This *Serratia* strain can stably colonize the gut of mosquito larvae and produce dsRNA to activate RNAi by effectively inhibiting the expression of growth- and development-related genes such as the methoprene-tolerant gene *Met* and the ecdysone receptor gene *EcR*, thus significantly inhibiting the development of mosquito larvae. More recently, Hu et al. developed a new strategy that employs multifunctional engineered symbiotic bacteria to suppress concurrent transmission of malaria parasites, as well as dengue and Zika viruses, by various vector mosquitoes [99], which presents a prominent prospect in future field applications for the control of mosquitoes and mosquito-borne diseases.

### 5.2. Gene Knockout

Gene knockout refers to a technique that inactivates or deletes specific genes in an organism through certain approaches. In studies related to insect gut microbiota, the homologous recombination-mediated and CRISPR-Cas9-mediated gene knockout methods are commonly adopted. The first replaces the target gene fragment with a designed homologous fragment (whose sequences at both ends are identical or highly similar to those of the target gene), thereby abrogating the function of the gene of interest. This method can also be used to insert specific elements into the genes of insects or microorganisms [100,101,102]. Furthermore, CRISPR-Cas9-mediated gene knockout enables precise recognition and binding to the target gene DNA sequence via sgRNA, which guides the Cas9 nuclease to cleave the double-stranded DNA at the target locus [103,104]. Compared with RNAi, gene knockout technology operates on DNA rather than RNA; its knockout effect is permanent, the target gene completely loses its function, and the resulting effects are heritable.

To analyze the promotion mechanisms of the commensal bacterium *Enterobacter cloacae* N29 for the development of the host insect *B. dorsalis*, *pdxA* gene (the key gene involved in vitamin B6 synthesis) knockout mutants of *E. cloacae* N29 were constructed using the homologous recombination system [100]. The results showed that the *pdxA* gene knockout strain lost the ability to promote larval growth, while supplementation with vitamin B6 could rescue the growth phenotype of axenic larvae, which indicated that the gut microbiota can promote insect growth and development by synthesizing vitamin B6. Furthermore, gene deletion mutants of *Pseudomonas fulva* ZJU1, a gut bacterium of silkworm, were constructed using the CRISPR-Cas9 technique to analyze the bacterial detoxification mechanism in 1-Deoxynojirimycin (DNJ), a mulberry-derived secondary metabolite that is toxic to most Lepidopteran insects [102]. Following the knockout of the key gene *ilvB*, *P. fulva* ZJU1 was unable to metabolize DNJ, indicating that the thiamine pyrophosphate-binding protein encoded by *ilvB* is the core factor for DNJ degradation.

## 6. Functions of Insect Gut Bacteria Revealed by Integrated Multi-Techniques

**Figure 1:**
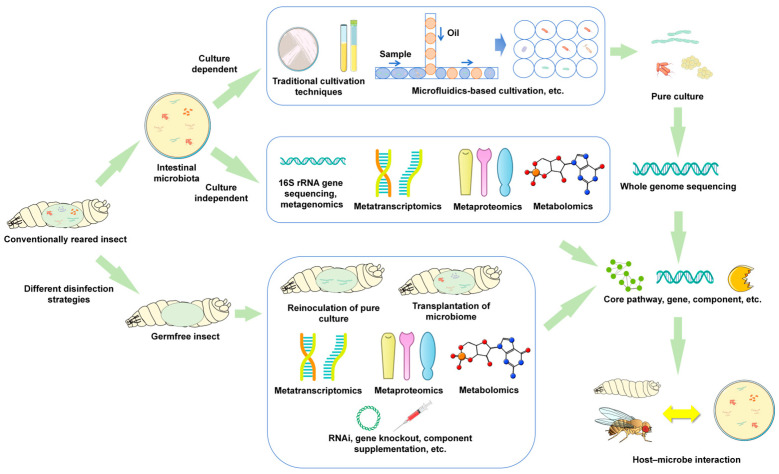
Integrated multi-technique strategies for the study of insect gut microbiota.

The integration of pure culture techniques with the germfree insect system has enabled extensive research on the functions of insect gut microbiota. Combined with meta-omics and other relevant research technologies, various research strategies have been designed and implemented for the study of gut microbial diversity and functions, which has greatly promoted the understanding of the impacts of gut microbiota on their hosts. Axenic and gnotobiotic insects serve as simple models for the analysis of intestinal microorganisms. Studies based on these systems have gained an understanding of the functions, as well as the transmission and variation in intestinal microbiota in hosts. The application of integrated multi-techniques with both culture-dependent and -independent strategies is common in studies of gut microbiota, as summarized in Figure 1. The following sections provide examples of functional analyses of insect gut microbiota through integrated multi-techniques.

### 6.1. Contributions to the Host Insect Nutrition

Previous studies have demonstrated that the gut microbiome of insects contributes to their host nutrition by producing a variety of digestive enzymes for food processing and essential substrates such as vitamins and amino acids [24,103,104,105]. A detailed study with an integrated multi-technique strategy illustrated that symbiotic bacteria with different carbohydrate-active enzymes were crucial for plant polysaccharide digestion in their corresponding hosts [52]. In this study, Vera Ponce de León and coworkers analyzed the symbiont-aiding mechanisms of the omnivorous American cockroach, *P. americana*, which thrives on a diet rich in plant polysaccharides, with 16S rDNA amplicon sequencing, pure cultures and whole-genome sequencing (WGS). Under anaerobic conditions, 11 strains belonging to the phylum Bacteroidetes were isolated from the digestive tract of *P. americana*. Phylogenetic and genomic analyses confirmed that these strains represent novel species of the genera *Bacteroides*, *Dysgonomonas*, *Paludibacter*, and *Parabacteroides*. Whole-genome sequencing indicated that the genomes of all these strains contain genes encoding carbohydrate-active enzymes (CAZymes), and some are also capable of mediating the extracellular secretion of these enzymes. In vitro culture and enzymatic activity experiments verified that certain strains can degrade starch, pectin, and/or cellulose and grow with these substrates as the sole carbon source. These findings revealed that the Bacteroidetes symbionts employ diverse polysaccharide-degrading strategies to help the host utilize plant polysaccharide-rich diets.

Another study with integrated multi-techniques demonstrated that *Drosophila*-associated bacteria shaped the nutritional requirements of their host during juvenile growth [106]. Through genomic analysis and metabolic network reconstruction of *D. melanogaster* and its gut bacteria, Consuegra and coworkers found that germfree *D. melanogaster* larvae cannot independently synthesize B vitamins and ten essential amino acids. In contrast, the isolated gut bacteria *Acetobacter pomorum* WJL (Ap^WJL^) and *Lactobacillus plantarum* NC8 (Lp^Nc8^) are capable of partially synthesizing the mentioned required nutrients, and their nutrient synthesis capacities were verified through in vitro culture experiments using specific media. Through bacteria–larva symbiosis experiments and the establishment of heat-killed (HK) bacteria control groups, it was discovered that most nutrient compensation requires metabolic processes of live bacteria, while a few nutrients can be partially compensated for by trace nutrients accumulated in HK bacteria. Quantitative analysis of the metabolites from bacterial cultures further confirmed that these two strains regulate the host’s nutritional requirements by directly synthesizing nutrients, providing metabolic intermediates, or supplying trace elements.

### 6.2. Regulation of Host Metabolic Pathways

To elucidate the molecular mechanisms by which the gut microbiota regulates host metabolism, germfree insects are commonly used as the core models. Research is typically performed via single-strain recolonization or microbiota transplantation, coupled with meta-omics screening, qPCR quantification, physiological index validation, and strategies such as gene interference and pathway inhibition.

Queen and worker bees are genetically identical but display dramatic divergence in lifespan. 16S rRNA gene sequencing has revealed marked differences in their gut microbial communities. Using microbiota-free (MF) worker bees, Wang and coworkers explored the molecular mechanisms underlying lifespan extension by transplanting queen gut microbiota to MF worker bees [92]. The queen-origin gut microbiota (OG) significantly prolonged the lifespan of worker bees. Metabolomic analysis identified lifespan-related differential metabolites, enriched in three pathways: insulin/insulin-like growth factor signaling (IIS), immunity, and ketone body metabolism. Immunological analysis indicated that OG does not regulate lifespan through the immune pathway. Supplementation of differential ketone bodies in MF workers did not extend lifespan, excluding ketone body metabolism from lifespan modulation. qPCR analysis further revealed that OG downregulated insulin-like peptides (ILPs) and upregulated the expression of antioxidant genes. This work ultimately illustrates that the queen’s gut microbiota extends host lifespan by suppressing IIS and strengthening antioxidant defense.

A recent study conducted by Han and coworkers elucidated the mechanism by which the gut microbiota modulates honey bee metabolism under a high-sugar diet, using both culture-independent and culture-dependent techniques [107]. By comparing microbiome-depleted (MD) bees, CL bees (those with five core gut genera), and conventional bees (CV), the authors found that gut microbiota depletion induced Type 1 diabetes-like symptoms in humans, including hyperglycemia, impaired lipid storage, and decreased metabolism. Reinoculation of the gut bacterium *Lactobacillus* Firm-5 into MD bees alleviated these symptoms. Comparative analysis of MD and CL bees with LC-MS, transcriptomics, and qPCR revealed that *Lactobacillus* Firm-5 can produce succinate via the reversed TCA cycle, activate the gluconeogenesis pathway, and promote the expression of insulin-like peptide genes (*ilp1* and *ilp2*), which ultimately alleviates hyperglycemia and metabolic abnormalities in honey bees. RNAi-mediated knockdown was also performed of the *Fbp* gene, which encodes fructose-1,6-bisphosphatase (FBP)—the key rate-limiting enzyme of the gluconeogenesis pathway. The knockdown experiment resulted in a significant downregulation of *ilp* expression in honey bees from both the MD group and the Firm-5-inoculated group, which directly indicates that FBP-mediated intestinal gluconeogenesis is an essential prerequisite for *ilp* activation.

### 6.3. Assistance in Host Defense Against Pathogens

Insects eliminate exogenous pathogenic microorganisms while maintaining the balance of symbiotic bacteria through physical defense (intestinal structure, peritrophic matrix, and epithelial cells), three major immune mechanisms (the immune deficiency (Imd)-signaling pathway, the dual oxidase–reactive oxygen species (Duox-ROS) system, and the JAK/STAT pathway), and interactions with intestinal symbiotic microbiota [108]. The intestinal symbiotic bacteria in insects help regulate and stabilize the intestinal environment [24] and generally resist pathogen invasion by regulating host immune pathways [109] or the interactions between microbial communities. Studies have shown that the gut microbiota confers higher host tolerance to bacteria, viruses, and Plasmodium parasites [109,110,111]. To reveal the specific role of the gut microbiota in host–pathogen resistance, pure cultured bacterial strains are used to verify their antipathogenic activity through in vivo and in vitro experiments, and RNAi or gene knockout techniques are frequently applied to further explore and validate the specific immune pathways or key genes related to antipathogenic substances.

One example is the work of Bahia and coworkers, who elucidated the anti-Plasmodium activity of *Anopheles* gut bacteria with integrated techniques including bacterial colonization assays, in vivo and in vitro *Plasmodium* inhibition assays, qPCR and RNAi-based immune pathway analysis, and *Anopheles* survival monitoring [109]. The gut microbiota can regulate the immune pathway to enable mosquitoes to resist bacteria, viruses, and plasmodia. In in vivo experiments, aseptic mosquitoes obtained with antibiotic treatment were fed a blood meal containing different bacterial strains and *Plasmodium*, with the aseptic group as a control. By detecting the number of *Plasmodium* oocysts in the intestinal tract, it was found that all the aforementioned strains could completely block infection. In in vitro experiments, *Plasmodium* was co-cultured with bacteria, and the fluorescence-labeled *Plasmodium* ookinetes were detected. Results showed that all the aforementioned strains could inhibit ookinete development. Transcription analysis and qPCR revealed that these gut bacteria could upregulate the expression of Imd pathway-related genes in *Anopheles* mosquitoes to varying degrees. Furthermore, after silencing the Imd pathway via RNAi, the anti-*Plasmodium* activity of the strains was weakened and the infection rate increased, confirming that gut bacteria can exert anti-*Plasmodium* effects through the Imd pathway. Among these bacteria, a *Serratia marcescens* isolate exhibits excellent gut colonization ability and anti-*Plasmodium* activity.

The gut microbiota itself can also inhibit pathogenic microorganisms, as proven by many bacteria isolated from different insects [112,113]. Using biochemical analysis, molecular biological identification, and gene functional verification, researchers confirmed that bacteriocins are the key active substances responsible for the antibacterial activity of the strains [114]. Antibacterial experiments have shown that bacteria isolated from the gut of honey bee larvae can inhibit various pathogens. Among them, *Enterococcus faecium* SM21 exhibits a specific inhibitory effect on *Listeria monocytogenes*. Treatment of the cell-free supernatant (CFS) of SM21 with different enzymes revealed that only the antibacterial activity of the protease-treated group disappeared, indicating that the antibacterial substance is a peptide or protein (consistent with the core characteristics of bacteriocins). Through SDS-PAGE and PCR amplification of enterocin-encoding genes, it was found that the molecular weight of this antibacterial substance was completely consistent with that of enterocins. Thus, the researchers concluded that *E. faecium* SM21 can produce bacteriocin-like substances to inhibit pathogenic bacteria.

### 6.4. Assisting Hosts in Degrading Toxic Compounds

Insects often inhabit environments containing antibiotics, insecticides, plant secondary metabolites, or other harmful substances that impede their growth, whereas gut microbiota can assist the host in surviving in such an environment. The mechanisms of toxic compound degradation by intestinal bacteria were elucidated with the application of multiple techniques [99].

Through whole-genome sequencing and analysis of *Burkholderia* SFA1, a gut symbiotic bacterium isolated from *Riptortus pedestris* (Hemiptera: Alydidae), Hu and coworkers obtained relevant information about the gene cluster responsible for the degradation of the insecticide fenitrothion (MEP) in the strain [99]. Comparative metatranscriptomic analysis was conducted for in vitro (SFA1 cultured in MEP-containing medium) and in vivo (SFA1 inoculated into larval bodies) systems. The results showed that the gene encoding the methyl parathion-degrading enzyme (*mpd*) was the key gene in SFA1 that mediates MEP degradation and confers insecticide resistance to the host. By knocking out the *mpd* gene to construct the *mpd*-complemented mutant, the researchers assayed the growth performance of the mutants and the complemented strain in the minimal medium with MEP as the sole carbon source, as well as the host survival rate upon MEP exposure when *R. pedestris* was inoculated with the strains. Meanwhile, HPLC was performed for the analysis of the metabolites to evaluate the MEP degradation efficiency of the in vitro-cultured bacteria and the isolated insect midguts. These experiments collectively verified that the *mpd* gene is the essential and sufficient key gene that mediates MEP degradation by the symbiont and endows the host with insecticide resistance, which converts MEP into a non-toxic compound for the host.

Plant secondary metabolites (e.g., glucosinolates, alkaloids, and terpenoids) defend against herbivores through mechanisms such as membrane disruption, nutrient and ion transport inhibition, and metabolism inhibition. Many insects have established symbiotic relationships with gut bacteria, which help them metabolize and detoxify these secondary metabolites, thereby enhancing the ability to acquire nutrients from plants [115,116,117,118]. Zhang et al. elucidated the mechanism by which silkworm gut bacteria degrade plant secondary metabolites with comprehensive techniques including 16S rRNA sequencing, strain isolation, multi-omics analysis, gene editing, and in vitro and in vivo functional validation, using the silkworm–mulberry system as a model [102]. 1-Deoxynojirimycin (DNJ) is a mulberry-derived secondary metabolite that is toxic to most lepidopteran insects. However, the silkworm, which feeds on mulberry leaves, can grow normally. 16S rRNA gene amplicon analysis revealed that high concentrations of DNJ altered the structure of the silkworm gut microbiota. Genomic and transcriptomic analyses indicated that *Pseudomonas fulva* ZJU1, isolated from the silkworm gut, has the potential to degrade DNJ. In vitro and in vivo DNJ degradation experiments demonstrated that *P. fulva* ZJU1 can grow using DNJ as the sole carbon source, and only live *P. fulva* ZJU1 can significantly enhance insects’ resistance to DNJ. UPLC-MS analysis clarified the metabolic pathway of DNJ. CRISPR-Cas9 technology was used to construct relevant gene deletion strains, which further verified the DNJ degradation mechanism of *P. fulva* ZJU1 and the key gene involved in the degradation pathway (the *ilvB* gene). In addition, inoculating *P. fulva* ZJU1 into other lepidopteran insects can significantly enhance their resistance to DNJ and restore their growth, indicating that the strain can broadly improve the resistance of lepidopteran insects to DNJ.

## 7. Discussion

In this review, we summarize the research strategies concerning insect gut microbiota. In general, the integration of multiple techniques with both culture-dependent and culture-independent methods is practical for most of the studies, which includes the following approaches: (1) exploring microbial diversity and functional potential using SSU rRNA gene amplicon sequencing or metagenomic sequencing; (2) isolating intestinal microorganisms and performing whole-genome sequencing; (3) identifying the functions of pure cultured microbes by reinoculating them to axenic insects, and exploring different phenotypes among axenic, gnotobiotic, and conventional insects with meta-omics techniques; (4) identifying key genes and metabolic pathways by combining metatranscriptomics with qPCR validation, metabolomics, and/or metaproteomics; (5) verifying the function of key genes through RNAi and/or gene knockout; and (6) further validating functional characteristics through in vivo and in vitro experiments. Based on different research objectives, further modifications and supplements to the strategies are encouraged. It should be emphasized that this review selects only typical research strategies and a few case studies on insect gut microbiota for discussion. The techniques and strategies used for these studies are also applicable in studies on the functions of the gut microbiota in rodents, fish, mammals, and other animals. It is found that the gut microbiota of different species shares functional similarities (such as regulating development, neural activity, and immunity), and research strategies targeting different species can be mutually referenced [119].

Although the development and application of integrative techniques have greatly promoted the functional analysis of insect gut microbiota and the interpretation of host–microbe interactions, there are still challenges that need to be confronted. With the combination of different strategies, many insect gut bacteria have been isolated and functionally analyzed. However, most intestinal anaerobes, including fastidious bacteria and archaea, still remain uncultured. The in-depth analysis of microbe–insect interactions or interspecies relationships between microbes may offer hints for the development of new techniques to meet the growth conditions of those “hard-to-culture” microorganisms. At present, although the core microbial communities of a few insects (such as honey bees and termites) have been clarified [2,107], those of many other insects (such as black soldier flies) remain uncertain [120]. Further exploration and more strict rules or criteria are needed for the definition of the core microbiome, which is crucial for symbiont-based genetic modification [98]. Currently, most studies on axenic insect systems and strain reinoculation are still in a relatively preliminary stage [81]; axenic techniques have not been established for most insect species. Since the gut microbiota forms a complex community, the colonization mechanism of gut microorganisms and whether introducing only one or several isolated bacteria can fully reflect their true state in the host remain unclear. For the study of microbe–insect interactions, additional innovative techniques or new designs of multiple techniques are encouraged.

Synthetic biology is a newly developed discipline that combines biology and engineering to design and construct new biological parts, devices, and systems, or to design existing biological systems for useful purposes. Based on the understanding of insect–microbe interactions, it is possible to engineer components of the insect microbiome and introduce the synthetic microbial community (SynComs) into the insect gut, which can enhance pathogen resistance, nutrient provision, and host fitness [121]. The strategies for synthetic microbial community introduction have been proposed and challenges have been discussed, which lay a strong foundation for future work on the application of SynComs in pest management and beneficial insect conservation.

The research on insect–microbe interactions offers insights into the application of insect gut microorganisms in fields such as agriculture, health, and environmental management. The application in agriculture is reflected in the following aspects: For beneficial insects such as bees and silkworms, the elucidation of commensal gut microorganisms promotes the protection of these insects in the field, which will bring huge economic value [92,102]. For pests such as *B. dorsalis* and *Plutella xylostella* (Lepidoptera: Plutellidae), it is possible to inhibit their growth and development by regulating microbial homeostasis [108,118]. In the future, the application potential in agriculture will be further developed through behavioral regulation of insects or inoculating probiotics such as *Bacillus subtilis* in the field for pest control [122]. Applications in environmental management have also been explored in some insect species. Cellulose-degrading bacteria isolated from insects, as well as enzymes produced by these bacteria, have been used for the decomposition of agricultural wastes such as crop straw, manure, fruit, and vegetable residues. For instance, the gut bacteria of BSF larvae have enhanced the conversion efficiency of chicken manure and nutrient accumulation in larvae [123]. The powerful detoxification function of the insect gut microbiota enables it to degrade pollutants in agri-food waste, thus providing a guarantee for the safe application of insect-derived feed and frass fertilizer. In addition, the insect gut microbiota can inhibit the reproduction of pathogenic bacteria in substrates and reduce the potential of biosafety risks. In the meantime, the gut microbiota can synthesize essential amino acids for insects, ensuring insect growth in low-nutrient substrates and improving the protein content and quality of insect bodies [124]. The existing studies on microbe–insect interactions lay a strong foundation for applications in different fields such as health, including the control of mosquito-borne diseases as mentioned above [98,99].

In summary, further exploration of insect–microbiota interactions will enhance their application in diverse fields such as agriculture, health, and environmental management. New strategies, including synthetic biology technologies (such as engineered microorganisms and the assembly of synthetic microbial communities), hold significant research value and warrant further development.

## Data Availability

No new data were created or analyzed in this study. Data sharing is not applicable to this article.
